# Anti-cancer effect of snake venom toxin through down regulation of AP-1 mediated PRDX6 expression

**DOI:** 10.18632/oncotarget.4192

**Published:** 2015-06-01

**Authors:** Hye Lim Lee, Mi Hee Park, Dong Ju Son, Ho Sub Song, Jung Hyun Kim, Seong Cheol Ko, Min Jong Song, Won Hyoung Lee, Joo Hee Yoon, Young Wan Ham, Sang Bae Han, Jin Tae Hong

**Affiliations:** ^1^ College of Pharmacy, Medical Research Center, Chungbuk National University, Osong-eup, Heungduk-gu, Cheongju, Chungbuk, Republic of Korea; ^2^ Department of Acupuncture & Moxibustion Medicine, College of Korean Medicine, Gachon University, Bokjeong-dong, Sujeong-gu, Seongnam, Gyeonggii, Republic of Korea; ^3^ Department of Obstetrics and Gynecology, Daejeon St. Mary's Hospital, College of Medicine, The Catholic University of Korea, Jung-gu, Daejeon Republic of Korea; ^4^ Department of Nuclear Medicine Chungbuk National University Hospital, Seowon, Cheongju, Chungbuk Republic of Korea; ^5^ Department of Obstetrics and Gynecology, St. Vincent's Hospital, College of Medicine, The Catholic University of Korea, Paldal-gu, Suwon, Gyeonggi-do, Republic of Korea; ^6^ Department of Chemistry and Biochemistry, Brigham Young University, Provo, Utah, United States

**Keywords:** snake venom toxin, apoptosis, PRDX6, AP-1, lung cancer

## Abstract

Snake venom toxin (SVT) from Vipera lebetina turanica contains a mixture of different enzymes and proteins. Peroxiredoxin 6 (PRDX6) is known to be a stimulator of lung cancer cell growth. PRDX6 is a member of peroxidases, and has calcium-independent phospholipase A2 (iPLA2) activities. PRDX6 has an AP-1 binding site in its promoter region of the gene. Since AP-1 is implicated in tumor growth and PRDX6 expression, in the present study, we investigated whether SVT inhibits PRDX6, thereby preventing human lung cancer cell growth (A549 and NCI-H460) through inactivation of AP-1. A docking model study and pull down assay showed that SVT completely fits on the basic leucine zipper (bZIP) region of c-Fos of AP-1. SVT (0–10 μg/ml) inhibited lung cancer cell growth in a concentration dependent manner through induction of apoptotic cell death accompanied by induction of cleaved caspase-3, -8, -9, Bax, p21 and p53, but decreased cIAP and Bcl2 expression via inactivation of AP-1. In an xenograft *in vivo* model, SVT (0.5 mg/kg and 1 mg/kg) also inhibited tumor growth accompanied with the reduction of PRDX6 expression, but increased expression of proapoptotic proteins. These data indicate that SVT inhibits tumor growth via inhibition of PRDX6 activity through interaction with its transcription factor AP-1.

## INTRODUCTION

Lung cancer remains the most lethal malignancy in the world. Despite improvements in surgical treatment, systemic therapy, and radiotherapy, the 5-year survival rate for all patients diagnosed with lung cancer remains between 15 and 20% [1]. Non-small cell lung cancer (NSCLC) is the most common type of lung cancer, and can be further classified as nonsquamous carcinoma (including adenocarcinoma, which accounts for 40% of NSCLCs) and squamous NSCLC, which makes up 30% of NSCLC cases [2–4]. Inhibitors of the epidermal growth factor receptor (EGFR) are used for the treatment of NSCLC [5]. In addition, other inhibitors such as miR-99a and heat shock protein 90 (HSP 90) have been also involved in lung tumor development. Down-regulation of miR-99a is significantly associated with last-stage and tumor metastasis in NSCLC patients. Further functional experiments found that overexpression of miR-99a inhibit cell proliferation, migration and invasion of NSCLC cells *in vitro* and tumor metastasis of NSCLC *in vivo* [6]. HSP 90 is of considerable interest because tumor cells and oncogenic proteins are acutely dependent on its activity, and the HSP90 inhibitor is currently being clinically tested against a wide array of tumor cell lines, including lung cancer cell lines [7]. A proteomics analysis study suggests that the expression of cytokeratine 8, Y-box binding protein 1 (YB-1), proliferating cell nuclear antigen (PCNA), non-metastatic protein 23 (Nm23) were also significant in lung cancer development [8].

PRDX6, a 1-Cys PRDX, is a bifunctional protein that acts both as glutathione peroxidase and calcium-independent phospholipase A2 (iPLA2) [9, 10]. The mammalian PRDXs family is composed of six members, PRDX1–6. PRDXs 1–5 have two catalytically active cysteines, while PRDX6 is the sole 1-Cys member PRDXs function together to detoxify ROS and thus provide cytoprotection from internal and external environmental stress [11, 12]. A lot of research about correlation to the occurrence of cancer and the PRDXs family has been performed. Recent studies reported elevated expression of PRDX1 in several human cancers, including esophagus [13], breast [14] and prostate [15]. PRDX2 levels are increased in cervical cancer [16], colon cancer [17, 18] and metatstaic breast cancer in lung [19]. PRDX3 levels are increased in prostate cancer [20], lung cancer [21], breast cancer [22] and hepatocellular caricinoma [23]. PRDX4 levels are increased in glioblastoma cell [24], prostate cancer [25] and lung cancer [26]. PRDX5 is expressed in the thyroid gland where it could act as an antioxidant [27]. PRDX6 expression was significantly higher in human tissue samples of TSCCs (tongue squamous cell carcinomas) compared with the 10 corresponding adjacent normal tissues [28]. Other studies have shown the strong expression of PRDX2 and 3 isoforms in cervical intraepithelial neoplasia and cervical cancer [16]. Previously, we found that PRDX6 accelerates lung tumor progression via increased GPx and iPLA2 activities [29]. We also found that overexpression of PRDX6 promotes lung tumor growth via increased glutathione peroxidase and iPLA2 activities through the upregulation of the activating protein-1 (AP-1) and Jun N-terminal kinase (JNK) pathways [30].

The AP-1 complex is composed of homodimers of Jun family members (cJun, JunB and JunD), heterodi-mers of Jun and Fos (cFos, FosL1, FosL2, and FosB), or cAMP response element-binding protein (CREB)/activating transcription factor (ATF) family members [31, 32]. AP-1 stimulates genes involved in cancer cell invasion and metastasis, proliferation, differentiation, and survival [33, 34]. Of NSCLC patients, the expression of AP-1 in NSCLC was higher than that in normal lung tissues [35]. Recent studies reported that specific AP-1 blockade by the dominant negative c-Jun mutant, TAM67, inhibits the tumor number during the tumor promotion stage of lung tumorigenesis. Researchers used a transgenic mouse model directing conditional expression of TAM67 in lung epithelial cells to determine the effect of AP-1 inhibition on mouse lung tumorigenesis. [36]. Expression of Suppressor of AP-1, Regulated by IFN (SARI), as an AP-1 inhibitory protein expression in patients with NSCLC had a poor prognosis, and over-expression of SARI in A549 cells inhibited the growth and migration of these cells [37]. The human PRDX6, as an antioxidant enzyme, has an AP-1 binding sequence in the promoter region [38]. Thus, AP-1 is significant in the tumor preventing effect of PRDX6.

SVT of Vipera lebetina turanica is the substance derived from a natural product that has a diverse effects. SVT has an anti-inflammatory effect [39], anti-arthritic effect [40] and anti-cancer effect [41]. Previously, we demonstrated that SVT has an anti-cancer effect of prostate [42], ovarian [43], colon [44], lung cancer [45] and neroblostoma cell [46]. SVT is actually a group of basic peptides composed of 235 amino acids with six disulfide bonds formed by 12 cysteines [47] which binds to cysteine residues of target molecules. Our previous findings indicate SVT binds to the cysteines of NF-κB thereby blocking NF-κB activities [48]. This binding inhibited the tumor promoting capacity in prostate cancer cells [42]. Our previous study also showed that cysteine residue (C47) of PRDX6 directly binds to thiacremonone blunting the lung tumor promoting effect of PRDX [49]. Thus, it is possible that SVT binds to the cysteine residue of its transcription factor AP-1 in PRDX6, and as a result, the inactivation of PRDX6 inhibits lung cancer cell growth.

However, cancer cell growth inhibitory effects and possible mechanism of SVT in lung cancers has not been studied yet. In the present study, we evaluated anti-tumor effects of SVT in lung cancer cells through blunting AP-1 activity of PRDX6.

## RESULTS

### Effect of SVT on cell growth and apoptotic cell death in lung cancer cells

**Figure 1:**
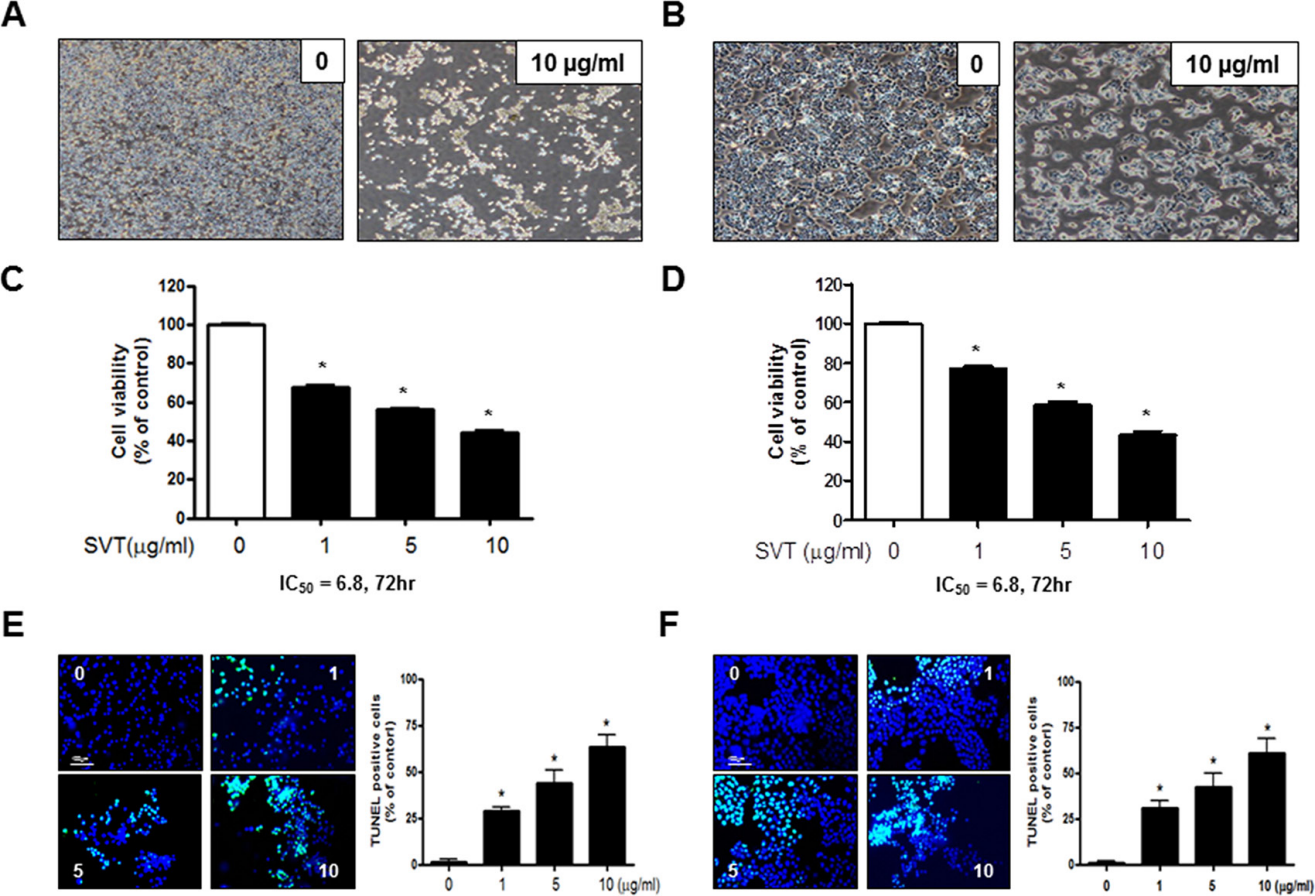
Effect of SVT on cell growth and apoptotic cell death in lung cancer cells Concentration-dependent effect of SVT on the MTT viability assay in A549 and NCI-H460 after 24 hr **C** and **D.** Morphologic observation with the treatment of SVT. A549 and NCI-H460 cells morphological changes were observed under phase contrast microscope **A** and **B.** respectively). The data were expressed as the mean ± S.D. of three experiments. *(*P* ≤ 0.05) indicates statistically significant differences from the control group. The lung cancer cells were treated with SVT for 24 hr, and then labeled with DAPI and TUNEL solution. Total number of cells in a given area was determined by using DAPI nuclear staining (fluorescent microscope). The green color in the fixed cells marks TUNEL-labeled cells. The apoptotic index was determined as the DAPI-stained TUNEL-positive cell number/total DAPI stained cell number (magnification, 200×) **E** and **F.** Values were means ± S.D. of three experiments. *(*P* ≤ 0.05) indicates statistically significant differences from the control cells.

To assess the inhibitory effect of SVT on cell growth of lung cancer cells; A549 and NCI-H460, we analyzed cell growth by MTT assay. Morphologic observation showed that the cells were gradually reduced in size and changed into a small round single cell shape with the treatment of SVT in A549 cells and NCI-H460 cells (Fig. 1A and 1B). The cells were treated with several concentrations of SVT (1, 5 and 10 μg/ml) for 72 hr. As shown in Fig. 1C and 1D, SVT inhibited growth of lung cancer cells in a concentration-dependent manner with IC_50_ value of 6.8 μg/ml in A549 cells, and IC_50_ values of 6.8 μg/ml in NCI-H460 cells, respectively. We performed DAPI staining followed by TUNEL staining assays, and then the double labeled cells were analyzed by a fluorescence microscope to determine the inhibition of cell growth by SVT was due to the induction of apoptotic cell death. Reversely, consistent with cell growth inhibitory effects, apoptotic cell death was significantly increased in SVT treated A549 and NCI-H460 lung cancer cells, respectively. The number of apoptotic cells (DAPI-positive TUNEL-stained cells) in A549 and NCI-H460 human lung cancer cell cultures was increased to about 62% and 73% of cells, respectively, at a concentration of 10 μg/ml (Fig. 1E and 1F).

### Structure of SVT and interaction between SVT and c-Fos of AP-1

**Figure 2:**
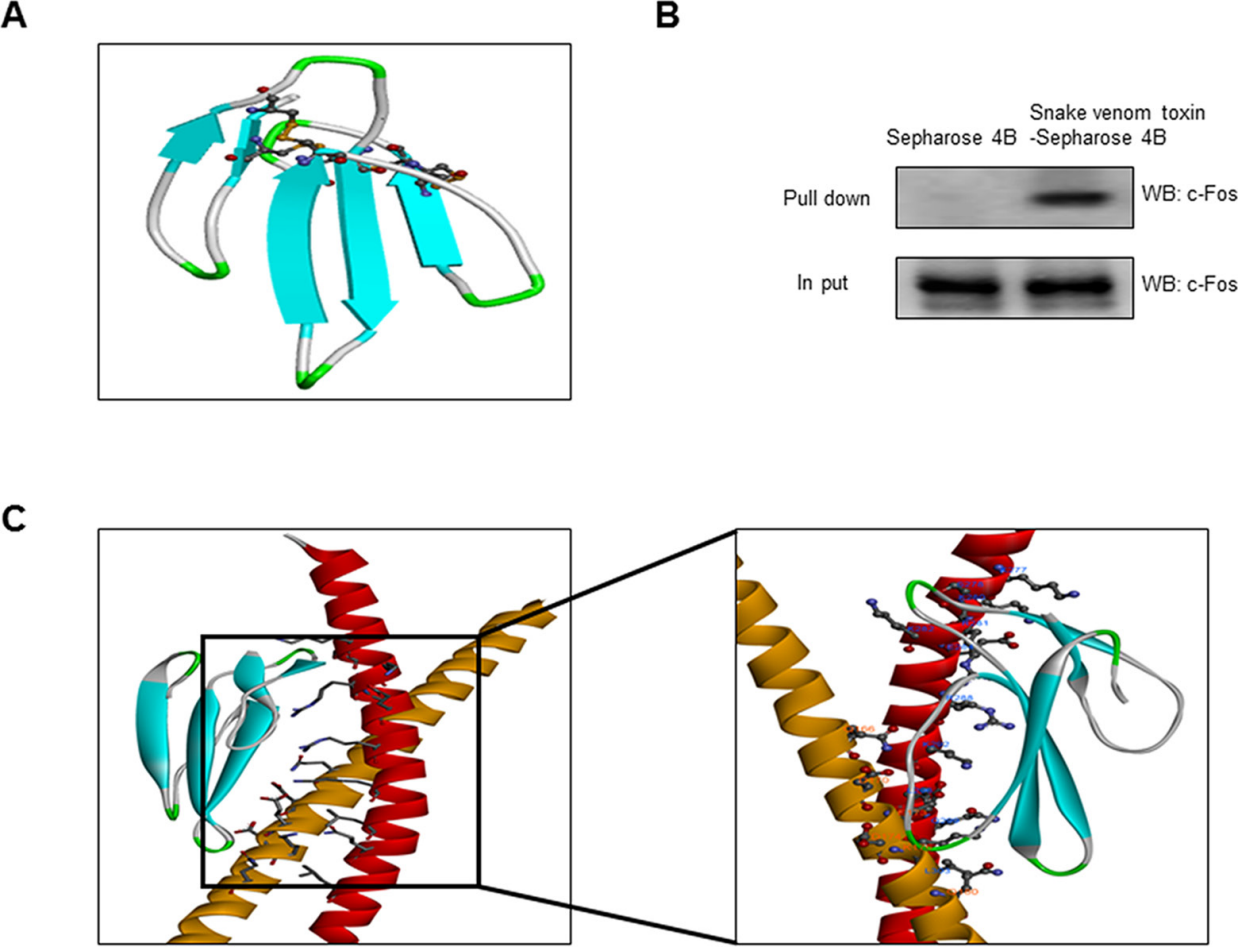
Structure of SVT and AP-1 and binding of SVT to c-Fos of AP-1 Structure of SVT of Vipera lebetina turanica. **A.** Structure of Cobrotoxin. **B.** Whole cell lysates of A549 were incubated with SVT-conjugated Sepharose 4B. After precipitation, the levels of bound c-Fos were monitored by Western blot analysis. **C.** Docking model of SVT with c-Fos. Molecular surface representation docking model of SVT with c-Fos. doi:10.1371/journal.pone.0091508.g001

The interaction of SVT (Fig. 2A)-Sepharose 4B beads with cell lysate containing c-Fos protein was assessed using a pull-down assay. The interaction of SVT-Sepharose 4B beads with c-Fos of AP-1 was then detected by immunoblotting with anti-c-Fos antibody. The results indicated that tectochrysin interacted with cell lysates containing c-Fos from A549 cells (Fig. 2B). To identify the binding site of SVT to c-Fos of AP-1, we performed computational docking experiments with SVT and c-Fos of AP-1. The best binding mode indicates that SVT binds in the basic leucine zipper (bZIP) region of AP-1. The binding pocket is comprised of Gln180, Lys176, Asp174, Glu173, Asp170 and Gln166 (Fig. 2C).

### Effect of SVT expression of apoptotic regulatory proteins

**Figure 3:**
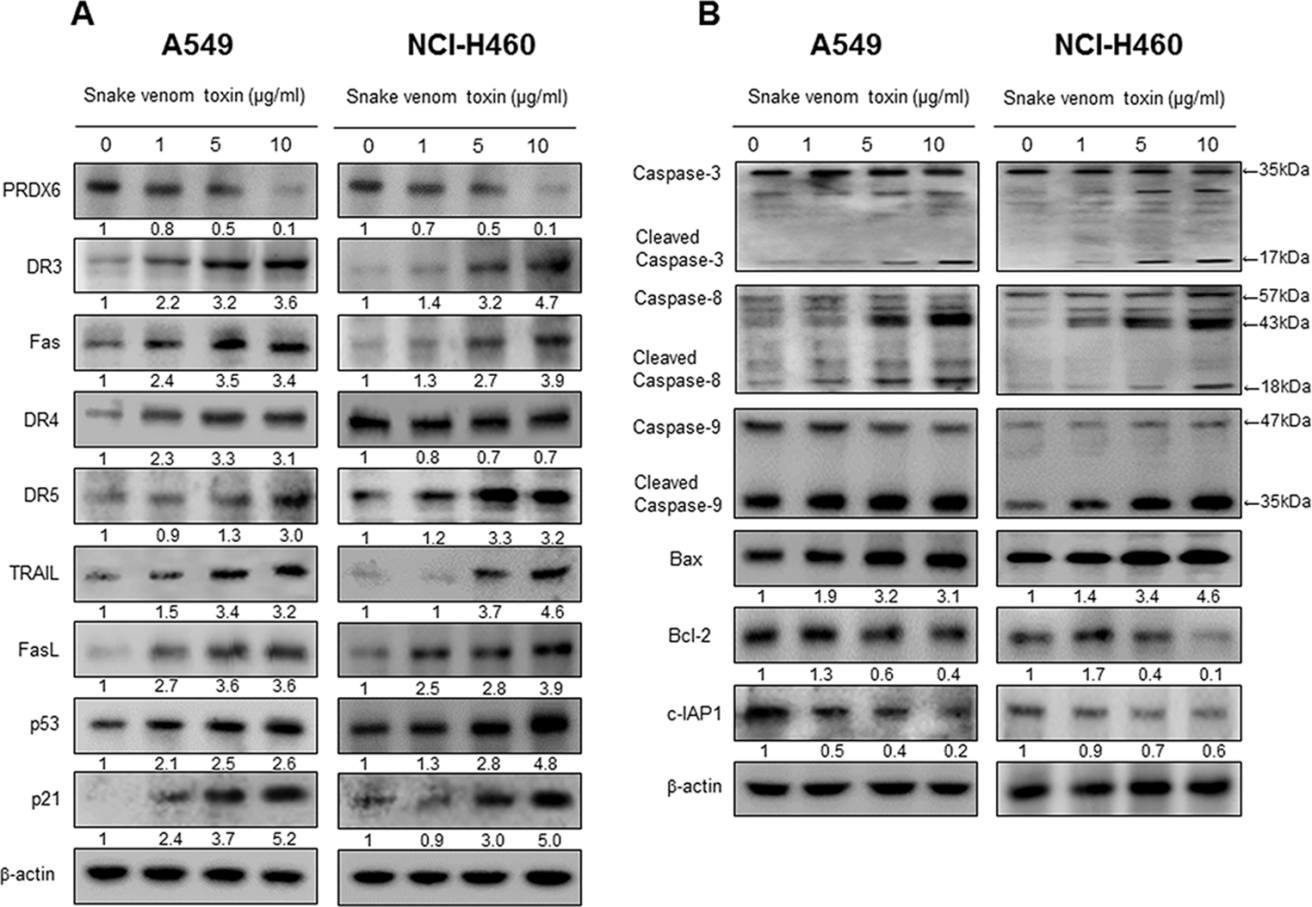
Effect of SVT expression of apoptotic regulatory proteins Expression of apoptosis regulatory proteins related exntrinsic pathway was determined using Western blot analysis with the antibodies against PRDX6, DR3, DR4, DR5, FAS, FASL, TRAIL, Bax, Bcl-2, c-IAP1, p53, p21, Caspase-3, caspase-8, caspase-9, and β-actin. β-actin protein was used an internal control. Each band is representative for three experiments **A** and **B.**

The activation of cell death regulatory proteins including DRs, caspases-3, -8 and -9 as well as Bax, leads to apoptosis in cancer cells. To figure out the expression of cell death regulatory proteins by SVT, the expression of apoptotic proteins was investigated by Western blots. The expression of pro-apoptotic proteins, Bax and cleaved form of caspase-3, -8, -9, and p21 and p53 were increased by a treatment of SVT. However, the expression of PRDX6, Bcl2, and c-IAP1 were decreased by the treatment of SVT in a concentration dependent manner (Fig. 3A and 3B).

### Effect of SVT on AP-1 activation

**Figure 4:**
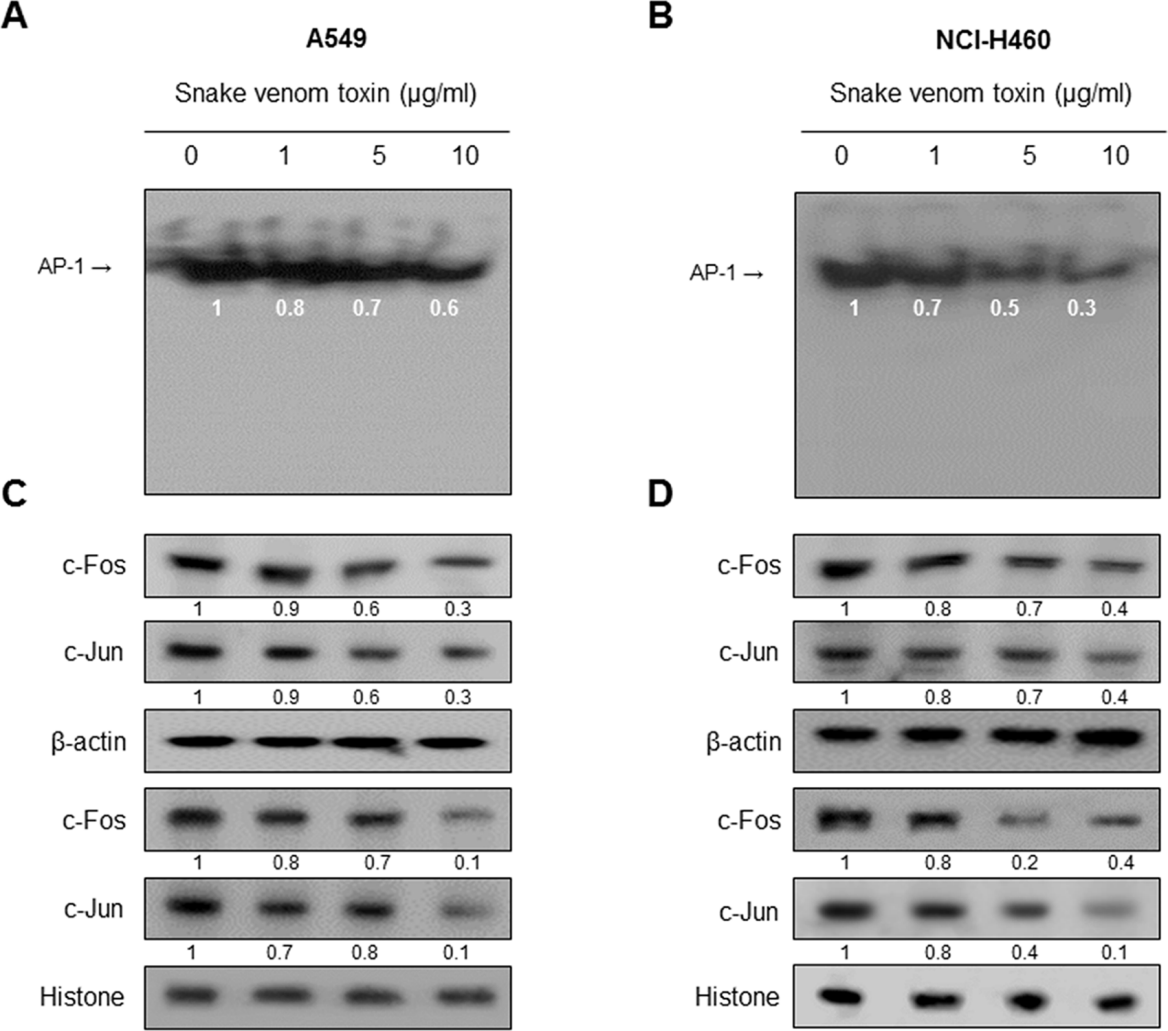
Effect of SVT on AP-1 activaion Nuclear extract from lung cancer cells treated with SVT (1, 5, and 10 μg/ml) for 2 hr was incubated in binding interaction of P^32^-end-labeled oligonucleotide containing the AP-1 sequence. The present EMSA results are representatives of three experiments **A** and **B**. The cells treated with SVT (1, 5 and 10 μg/ml) for 2 hr was incubated and were lysed, cytosolic proteins were used to determine the expression of c-Jun, c-Fos and β-actin (internal control) in lung cancer cells. Nuclear proteins were used to determine the expression of c-Jun, c-Fos and Histone (internal control) in lung cancer cells **C** and **D.** Each band is representative for three experiments.

We determined whether SVT can inhibit AP-1 DNA binding activity. Nuclear extracts from treated cells were prepared and assayed for AP-1 DNA binding by EMSA. Lung cancer cells have a strong AP-1 DNA binding activity, which was attenuated by the treatment of SVT in a concentration dependent manner (Fig. 4A and 4B). Consistent with the inhibitory effect on AP-1 activity, the expression in the nucleus protiens c-Jun and c-Fos and the cytosolic protiens c-Jun and c-Fos, components of AP-1, were also inhibited (Fig. 4C and 4D).

### Effect of SVT with AP-1 inhibitor (SR11302) and siRNA of c-Fos on the expression of PRDX6

**Figure 5:**
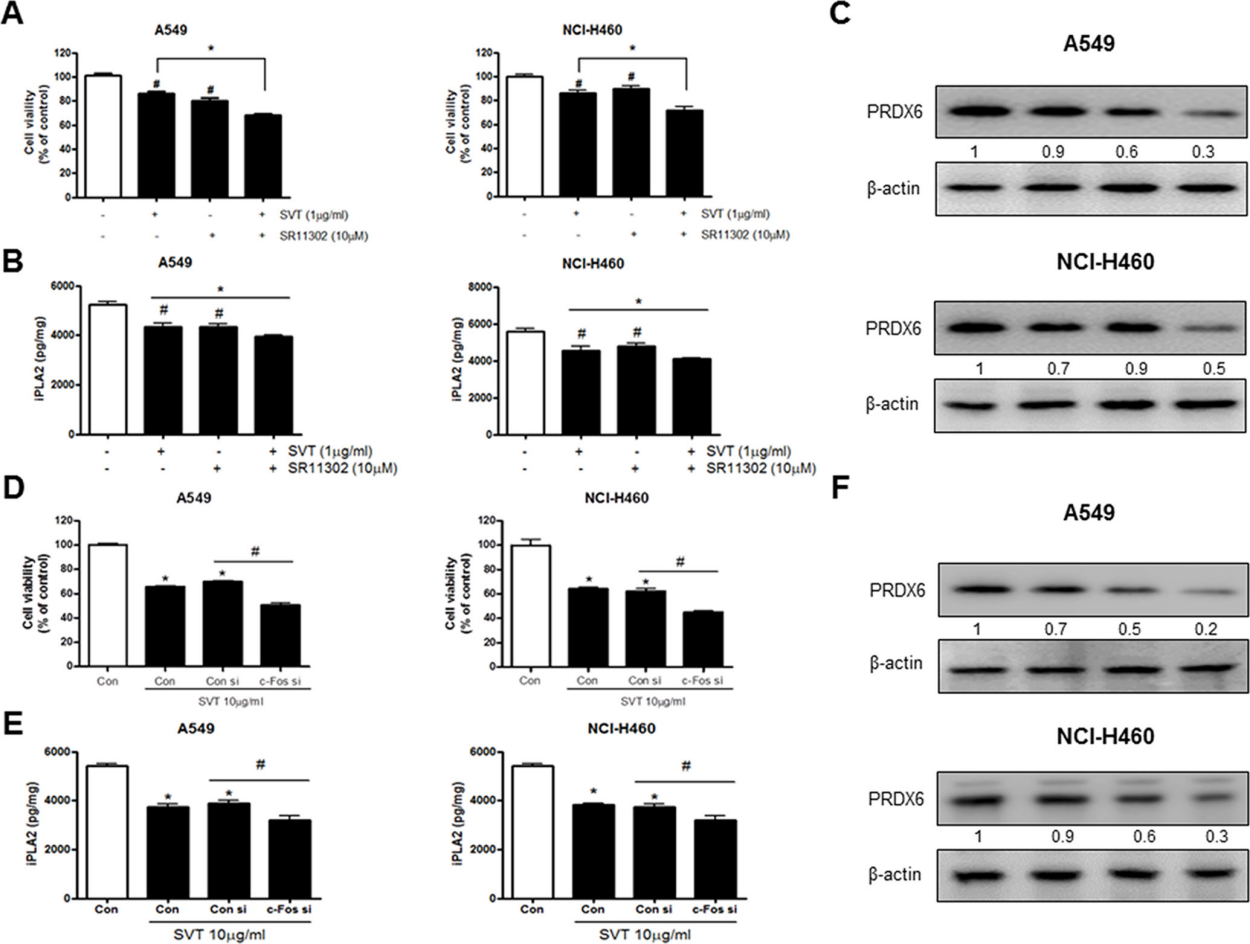
Effect of SVT with AP-1 inhibitor (SR11302) and siRNA of c-Fos on the expression of PRDX6 A549 and NCI-H460 cells were pretreated with AP-1 inhibitor (SR11302, 1 μM) for 30 min, the media were removed, and the cells were exposed to SVT (5 μg/ml) for 24 hr or 2 hr **A.** Cell viability was determined by MTT assay as described in Materials and Methods. Expression of iPLA2 was detected by ELISA kit **B.** Then equal amounts of total proteins (20 μg/lane) were subjected to 12% SDS-PAGE. Expression of PRDX6 and β-actin was detected by Western blotting using specific antibodies. β-actin protein was used an internal control **C.** The lung cancer cells were transfected with the c-Fos siRNA (100 nM) for 24 hr, the cells were then and treated with SVT (10 μg/ml) for 24 hr. After treatment, cell viability was measured by MTT assay **D.** Expression of iPLA2 was detected by ELISA kit **E.** Then equal amounts of total proteins (20 μg/lane) were subjected to 12% SDS-PAGE. Expression of PRDX6 and β-actin was detected by Western blotting using specific antibodies **F.** β-actin protein was used an internal control. Cell growths are means ± S.D. of three exprements. *(*P* ≤ 0.05) indicates statistically significant differences from control cells. #(*P* ≤ 0.05) indicates statically significant differences from SVT treated group.

To further demonstrate the involvement of AP-1 pathway in SVT-induced lung cancer cell growth, we compared the combination treatment of SVT and AP-1 inhibitor (SR11302, 10 μM) with SVT or AP-1 inhibitor alone. Cancer cells were pretreated with AP-1 inhibitor (SR11302, 10 μM) 30 min prior to the treatment of SVT (5 μg/ml), and then assayed cell growth and PRDX6 expression. The combination treatment of SVT with AP-1 inhibitor greatly inhibited lung cancer cell growth compared to those by SVT or AP-1 inhibitor alone in both cancer cells (Fig. 5A). We also found that a much lower expression of iPLA2 (Fig. 5B) and expression of PRDX6 (Fig. 5C) occured by the combination treatment of SVT and AP-1 inhibitor. To further determine the relationship between PRDX6 expression and lung cancer cell growth inhibitory effect of SVT, we transfected A549 and NCI-H460 cells with c-Fos siRNA using a transfection agent. The cells were transfected with 100 nM siRNA of c-Fos for 24 hr, and then treated with SVT (10 μg/ml) for another 24 hr. Knock down of c-Fos almost completely reversed the cell growth inhibitory effect of SVT in A549 and NCI-H460 (Fig. 5D). We also found a lower expression of iPLA2 (Fig. 5E) and expression of PRDX6 (Fig. 5F).

### SVT inhibited tumor growth in vivo xenograft

**Figure 6:**
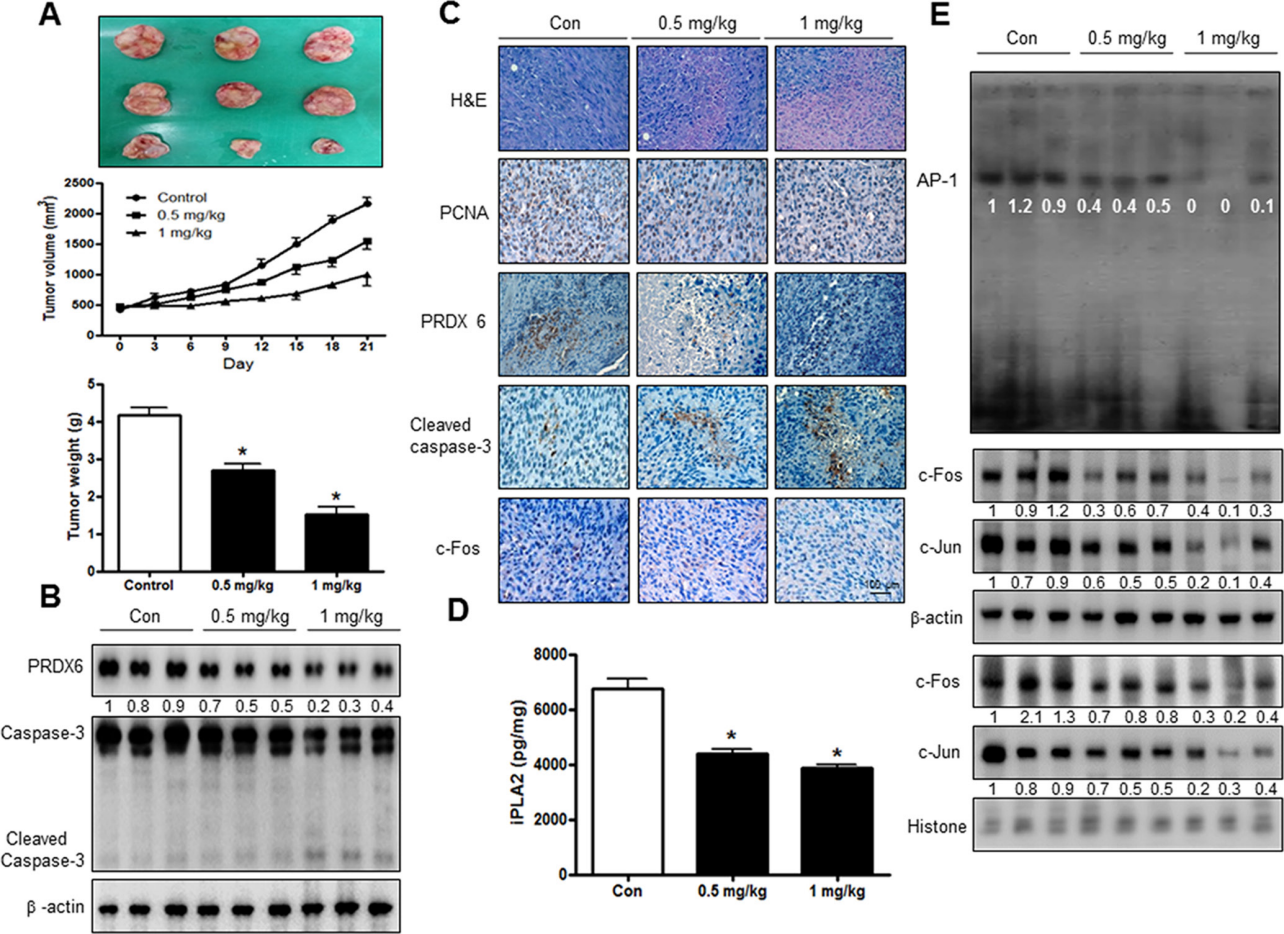
SVT inhibited tumor growth *in vivo* xenograft Tumor volumes, weights, and images of normal mice **A.** The expression of PRDX6 and Caspase-3 was detected by western blotting **B.** β-actin protein was used an internal control. Tumor sections of mice were analyzed by H&E, PCNA, PRDX6, Caspase-3 and c-Fos by immunohistochemistry **C.** Expression of iPLA2 was detected by ELISA kit **D.** AP-1 activity in tumor tissue **E.** The resultant tissues were developed with DAB, and counterstained with hematoxylin. Scale bar indicates 50 mm. *(*P* ≤ 0.05) indicates statistically significant differences from control cells.

To elucidate the anti-tumor effect of SVT *in vivo*, the tumor growth on lung cancer cell xenograft bearing nude mice following SVT (0.5 mg/kg and 1 mg/kg) treatments, was investigated. SVT (0.5 or 1 mg/kg injected intraperitoneally two times per every week for a period of 3 weeks) significantly inhibited tumor volume and tumor growth (Tumor volume and tumor weight, 30% or 40% over control by 1 mg/kg SVT) (Fig. 6A). Expression of pro-apoptotic proteins including cleavaged caspase-3 was concomitantly increased, but expression of PRDX6 was inhibited (Fig. 6B). Expression of PCNA, PRDX6 and c-Fos were inhibited, but pro-apoptotic proteins, including cleavaged caspase-3, were concomitantly increased (Fig. 6C). iPLA2 activity was also inhibited by the treatment with SVT (Fig. 6D). AP-1 activity and cytosol of c-Jun and c-Fos and nucleus translocation of c-Jun and c-Fos were inhibited in tumor tissues by the treatment with SVT (Fig. 6E).

## DISCUSSION

In the present study, we found that SVT inhibited cell growth of human lung cancer cells; A549 and NCI-H460 through the inhibition of PRDX6 activity via interaction to AP-1. SVT has an anti-inflammatory effect [39], anti-arthritic effect [40] and anti-cancer effect [41]. Previously, we demonstrated that SVT has an anticancer effect of prostate [42], ovarian [43], colon [44] and neroblostoma cells [46]. Our present study further demonstrated SVT could be a useful compound to treat lung cancer.

Our present findings showed that SVT inhibited lung cancer cell growth through the inhibition of PRDX6 activity via interaction to AP-1. A lot of research has been performed to demonstrate the relevance between PRDXs and tumor growth. PRDX6 overexpression attenuates cisplatin-induced apoptosis in human ovarian cancer cells; SKOV-3 [50]. Suppression of PRDX6 in Hepa1–6 cells would increase susceptibility to peroxide-induced cell death [51]. Previously, we found that PRDX6 accelates lung tumor progression [29]. Many compounds showed their anti-tumor activity by modification of PRDXs activity. Dioscin induces esophageal cancer cell apoptosis through downregulation of PRDX1 and 6 [13]. Multiple myeloma cell growth was selectively and significantly reduced by (−)-Epigallocatechin 3-gallate through the downregulation of PRDXs [52]. 4-amino-5-(4-chlorophenyl)-7-(t-butyl)pyrazolo[3, 4-d]pyrimidine (PP2) induces apoptosis and decreases the expression of PRDX3 in human breast cancer MCF-7 cells [22]. We also found that thiacremonone inhibited lung tumor growth in PRDX6 overexpressed transgenic mice through downregulation of PRDX6 [49]. These data suggest that downregulation of PRDX6 could be significant for SVT-induced lung cancer cell growth inhibition.

AP-1 stimulates genes involved in invasion and metastasis, proliferation, differentiation, and survival as well as angiogenesis [33, 34]. Of NSCLC patients, the expression of AP-1 in NSCLC was higher than that in normal lung tissues [35]. It was reported that inhibition of AP-1 by conditional expression of the dominant-negative c-Jun mutant in lung epithelial cells decreased tumor number and overall lung tumor burden in chemically induced mouse lung tumor models [36]. It was reported that the nucleotide sequence of the PRDX1 gene has potential AP-1 sites [38], and PRDX1 promoter region binds JunB and c-Fos of AP-1 [53]. Most studies related to identification of responsive elements in the PRDX6 gene promoter have described several redox-active transcription factors including a AP-1, suggesting that PRDX6 gene is also subjected to a complex transcriptional regulation through AP-1 [54]. Other studies also reported that promoter region of PRDX6 contains nucleotide sequence of AP-1 [38]. Previously, we found overexpression of PRDX6 promotes lung tumor growth via increased glutathione peroxidase and iPLA2 activities through the upregulation of the AP-1 and JNK pathways [30]. Knockdown of PRDX1 or PRDX4 significantly reduced the activation of c-Jun and thus repressed AP-1 mediated promoter activity, which may contribute to the changes of lung cancer cell phenotype [55]. Our previous study also found that higher lung tumor growth in PRDX6-overexpressing transgenic mice was associated with an increase in AP-1 DNA-binding activity [30]. Thus, inhibition of AP-1 by SVT could be significant for reduced PRDX6 activity, and thus decreased lung cancer cell growth. Our present results showed that expression of PRDX6 via inactivation of AP-1 in A549 and NCI-H460 lung cancer cell were decreased. However, treatment of c-Fos siRNA in A549 and NCI-H460 reversed SVT-induced lung cancer cell growth inhibition. We also found that PRDX6 expression was significantly lower in the SVT treated cultured human lung cancer cells as wells as xenograft tumor tissues. Lower expression of PRDX6, iPLA2 and greater cell growth inhibition occured in the combination treatment of AP-1 inhibitor (SR11302, 10 μM) with SVT compared to those by AP-1 inhibitor or SVT alone. Through the pull-down assay using SVT-agarose bead, we found that SVT bound with cell lysates containing c-Fos from human A549 lung cancer cells. We also found a direct interaction of SVT with c-Fos of AP-1 by the docking experiment. Other studies demonstrated that direct interaction of cyclopentenone 15-deoxy- Δ^12,14^-prostaglandin J_2_ (15d-PGJ_2_) with 269 cysteine of AP-1 protein, and thus contribute to the complex effects of 15d-PGJ_2_ on the cellular response to pro-inflammatory agents [56]. Arylstibonic acid NSC13746 binds specifically to c-Fos/JunD dimer of B-ZIP proteins at micromolar concentrations and can inhibit their DNA-binding activity both *in vitro* and *in vivo* [57]. Expression in the mouse epidermis of A-Fos, a dominant negative form that inhibits AP-1 DNA binding, converts papillomas into benign sebaceous adenomas that are not able to convert into carcinomas [58]. In *in vivo* study with A549 xenograft bearing mice, treatment of SVT (0.5 mg/kg and 1 mg/kg injected intraperitoneally twice a week for 3 weeks) significantly inhibited tumor growth by approximately 50–60%. The immunohistochemistry analysis of tumor section by H&E, and by proliferation antigens against PCNA staining revealed that SVT inhibited tumor growth. In addition, our data also showed that SVT inhibited expression of PRDX 6 and AP-1 activity in lung tumor tissues. Also, our data showed that SVT inhibited expression of AP-1 accompanied with inhibition of expression of iPLA2 in lung tumor tissues. Moreover, expression of proapoptotic proteins, cleaved form of caspase-3 and Bax, was increased and anti-apoptotic protein, but expression of Bcl2 was decreased by treatment of SVT. These data suggest that interaction of SVT with AP-1 block transcription of PRDX6 thus inhibiting PRDX6 could be implicated for lung tumor growth inhibition.

Snake venoms contain complex mixtures of pharmacologically active peptides and proteins and thus have a variety of pharmacological effects. Most significant effect is its anti-inflammatory effect through inhibition of NF-κB activities and its production of IL-1β, TNF-α, iNOS and CAM-1 [39]. It has anti-arthritic effect showing significant restoration in paw & ankle volume, paw weight, urinary hydroxyproline, glucosamine, serum ACP, ALP and IL-10 level by SVT treatment [40]. A recent study showed a protective effect of SVT against LPS-induced septic shock. Survival rate was significantly higher in SVT-treated rats, compared to that of non-treated septic rats. Furthermore, SVT treatment also significantly reduced LPS-associated TNF-α and LDH [59]. In addition, SVT has anti-tumor effects. SVT inhibited the proliferation, altered the cell cycle and enhanced the induction of apoptosis of breast cancer cells by increasing the activities of caspase-3, caspase-8 and caspase-9. Moreover, SVT sensitized the primary breast cancer cells to growth arrest and apoptosis by increasing the generation of free radicals, including reactive oxygen species (ROS), hydroperoxide and nitric oxide [60]. In our recent study, the anti-cancer effect of SVT in cervical cancer via increase of death receptor 3 and 5 and inactivation of NF-κB was demonstrated [61]. It was known that the LD_50_ value for SVT in mice is 2.5 mg/kg [62]. In a previous study [61] and in the present study, we observed that SVT (1 mg/kg) did not induce any serious health problems, such as eruption, swelling, weight loss, or death in the animals. Thus, the dosage of SVT for cancer treatment could be safe enough to develop as a drug. In conclusion, our result that natural toxin SVT could be useful as an anti-lung cancer agent through inhibiton of PRDX6 by the interaction with AP-1 which inhibited expression of PRDX6.

## MATERIALS AND METHODS

### Materials

Snake venom toxin from Vipera lebetina turanica was purchased from Sigma (St. Louis, MO). SR11302 was purchased from Tocris (Bristol, UK).

### Cell culture

The A549 and NCI-H460 lung cancer cell lines were obtained from American Type Culture Collection (ATCC). Cells were cultured in RPMI 1640 (Gibco, Life Technologies, Grand Island, NY) medium supplemented with 10% heat inactivated fetal bovine serum (FBS) and 100 units/mL penicillin, 100 μg/mL streptomycin (Invitrogen). Cell cultures were then maintained in an incubator within a humidified atmosphere of 5% CO_2_ at 37°C.

### Cell growth assay

Lung cancer cells, A549 and NCI-H460 cells, were plated in 96-well plates, and subsequently treated with SVT 0, 1, 5, 10 μg/mL for 24 hr. After treatment, cell viability was measured by MTT [3-(4, 5-Dimethylthiazol-2-yl)-2, 5-Diphenyltetrazolium Bromide] assay (Sigma Aldrich, St. Louis, MO) according to the manufacturer's instructions. Briefly, MTT (5 mg/mL) was added and plates were incubated at 37°C for 4 hr before 100 μL dimethyl sulfoxide (DMSO) was added to each well. Finally, the absorbance of each well was read at a wavelength of 540 nm using a microplate reader.

### Evaluation of apoptotic cell death

TUNEL assay was performed by using the DeadEnd^TM^ Fluorometric TUNEL System (Promega, Madison, Wisconsin, USA) for in situ detection of apoptotic cells, according to the manufacturer's instructions. Lung cancer cells (2 × 10^4^ cells/well) were cultured on 8-chamber slides, after cells were treated with SVT. The cells were washed with PBS and fixed by incubation in 4% paraformaldehyde in PBS for 20 min at room temperature. Membrane was permeabilized by exposure to 0.1% Triton X-100 in PBS for 5 min at room temperature. For DAPI staining, slides were incubated for 15 min at room temperature in the dark with a mounting medium for fluorescence containing DAPI (Vector Laboratories, Inc., Burlingame, CA). The cells were then observed through a fluorescence microscope (Leica Microsystems AG, Wetzlar, Germany). The total number of cells in a given area was determined by using DAPI and TUNEL staining. The apoptotic index was determined as the number of DAPI-stained TUNEL-positive cells divided by the total number of cells counted × 100.

### Western blotting

Lung cancer cells treated with SVT (0∼10 μg/mL) for 24 hr were homogenized with a protein extraction solution (PRO-PREPTM, Intron Biotechnology), and lysed for 60 min incubation on ice. The cell lysate was centrifuged at 15, 000 rpm for 15 min at 4°C. Equal amount of proteins (40 μg) were separated on a SDS/12%-polyacrylamide gel, and then transferred to a polyvinylidene difluoride (PVDF) membrane (GE Water and Process technologies). Blots were blocked for 1 h at room temperature with 5% (w/v) non-fat dried milk in Tris-Buffered Saline Tween-20 [TBST: 10 mM Tris (pH 8.0) and 150 mM NaCl solution containing 0.05% Tween-20]. After a short washing in TBST, the membranes were immunoblotted with the following primary antibodies: caspase-3, caspase-9, caspase-8, c-IAP1 and Bcl-2 (1:1000 dilutions; Cell Signaling, Beverly, MA) and c-Jun, c-fos, p21, p53, DR3, DR4, DR6, Fas, TRAIL, Fas Ligand and (1:2000 dilutions; Santa Cruz Biotechnology, Santa Cruz, CA) and DR5, PRDX 6 (1:1000 dilutions; Abcam, Cambridge, UK) The blots were performed using specific antibodies followed by second antibodies and visualization by a chemiluminescence (ECL) detection system.

### Electro mobility shift assay

The DNA binding activity of NF-κB was determined using an electrophoretic mobility shift assay (EMSA) performed according to the manufacturer's recommendations (Promega). In short, A549 and NCI-H460 cells were cultured on 100-mm culture dishes. After treatment with SVT for 2 hr, the cells were washed twice with PBS, followed by the addition of 1 ml of phosphate buffered saline (PBS), and then the cells were scraped into a cold Eppendorf tube. Nuclear extracts were prepared and processed for EMSA as previously described. The relative densities of the DNA-protein binding bands were scanned by densitometry using MyImage (SLB), and quantified by Labworks 4.0 software (UVP, Inc., Upland, CA).

### Transfection of siRNA

Lung cancer cells (1 × 10^4^ cells/well) were plated in 96-well plates and transiently transfected with c-Fos siRNA, using a mixture of siRNA and the WellFect-EX PLUS reagent in OPTI-MEN, according to the manufacturer's specification (WelGENE, Seoul, Korea). The transfected cells were treated with 10 μg/ml SVT for 24 hr or 1 hr and then used for detecting cell viability and protein expression (1 hr culture) and AP-1 activation (1 hr culture).

### Docking procedure

The docking of AP-1 transcription factor with cobrotoxin was performed using a rigid-body docking program ZDOCK 3.0.2 on ZDOCK server (http://zdock.umassmed.edu) [63]. ZDOCK server allows easy and fast production of structural models of protein-protein complexes. AP-1 transcription factor from PDB ID:1FOS was used for the docking. Only one set of fos-jun heterodimer was selected and the duplex DNA bound to fos-jun heterodimer was not included in the docking experiments to allow full search of binding interface for the other protein counterpart. Only one monomer of Cobrotoxin was selected from PDB ID: 1V6P. Docking experiments were performed without selection or blocking of residues.

### Pull down assay

SVT was conjugated with cyanogen bromide (CNBr)-activated Sepharose 4B (Sigma-Aldrich, St. Louis, MO). Briefly, SVT (1 mg) was dissolved in 1 ml of coupling buffer (0.1 M NaHCO_3_ and 0.5 M NaCl, pH 10. The CNBr-activated Sepharose 4B was swelled and washed in 1 mM HCl through a sintered glass filter, then washed with the coupling buffer. CNBr-activated Sepharose 4B beads were added to the SVT-containing coupling buffer and incubated at 4°C for 24 hr. The SVT-conjugated Sepharose 4B was washed with three cycles of alternating pH wash buffers (buffer 1, 0.1 M acetate and 0.5 M NaCl, pH 4.0; buffer 2, 0.1 M Tris-HCl and 0.5 M NaCl, pH 8.0). SVT-conjugated beads were then equilibrated with a binding buffer (0.05 M Tris-HCl and 0.15 M NaCl, pH 7.5). The control unconjugated CNBr-activated Sepharose 4B beads were prepared as described above in the absence of SVT. The cell lysate was mixed with SVT conjugated Sepharose 4B or Sepharose 4B at 4°C for 24 hr. The beads were then washed three times with TBST. The bound proteins were eluted with SDS loading buffer. The proteins were then resolved by SDS-PAGE followed by immunoblotting with antibodies against c-Fos (1:2000 dilutions; Santa Cruz Biotechnology, Santa Cruz, CA).

### Measurement of iPLA2

Lysate of tumor tissue were used. Lysate of tumor tissue were obtained through protein extraction buffer containing protease inhibitor. iPLA2 levels were determined using each specific ELISA Kit (Cloud Clone Corp.). In brief, 100 μl of sample was added into a precoated plate and incubated overnight at 4°C. After washing each well of the precoated plate with a washing buffer, 100 μl of labeled antibody solution was added, and the mixture was incubated for 1 hr at 4°C in the dark. After washing, chromogen was added, and the mixture was incubated for 30 min at room temperature in the dark. Finally, the resulting color was assayed at 450 nm using a microplate absorbance reader (Sunrise^TM^, Tecan, Switzerland) after adding stop solution.

### Animal experiment

To conduct *in vivo* studies, male BALB/c nude mice (aged 6–7 weeks, weighing 20–25 g) were used. Nude mice were housed under specific pathogen free conditions according to the guidelines of the Animal Care Committee at the Chungbuk National University (CBNU-278-11-01). On day 0, A549 cells in PBS (2 × 10^7^ tumor cells/0.1 ml PBS/ani-mals) were injected subcutaneously into nude mice. The mice were divided into three groups (*n* = 6). SVT (0.5 mg/kg and 1 mg/kg) was administrated intraperitoneally twice per week for 3 weeks to mice with tumors ranging from 100 to 300 mm^3^. Tumor volumes were estimated by the formula: length (mm) × width (mm) × height (mm)/2 at the end of experiment.

### Immunohistochemistry

All tissues were fixed in 4% paraformaldehyde and cut into 4 μm sections using a freezing microtome. The sections were stained with hematoxylin and eosin (H&E) for pathological examination. For immunohistological staining, tumor sections were incubated in primary antibody. After rinse in phosphate buffered saline (PBS), the sections were subject to incubation in biotinylated secondary antibody. After the slides were washed and developed with DAB, the slides were counterstained with hematoxylin, mounted in aqua-mount, and evaluated on a light microscope (Olympus, Tokyo, Japan). Sections were dehydrated in a series of graded alcohols, cleared in xylene and coverslipped using Permount (Fisher Scientific, Suwanee, GA).

### Statistical analysis

The data were analyzed using the GraphPad Prism 4 ver. 4.03 software (GraphPad Software). Data were presented as mean ± S.D. The differences in all data were assessed by one-way analysis of variance. When the *p* value in the ANOVA test indicated statistical significance, the differences were assessed by the Dunnett's test. A value of *p* ≤ 0.05 was considered to be statistically significant.
